# Estimation of Appendicular Skeletal Muscle Mass in Studies Based on CHARLS May Cause Unreliable Conclusion

**DOI:** 10.1002/jcsm.13800

**Published:** 2025-04-16

**Authors:** Mingchong Liu, Jiaming Wang, Chensong Yang, Guixin Sun

**Affiliations:** ^1^ Department of Traumatic Surgery Shanghai East Hospital, School of Medicine Tongji University Shanghai China

We write to highlight a potential issue in the estimation of appendicular skeletal muscle mass (ASM) in studies based on the China Health and Retirement Longitudinal Study (CHARLS). In recent years, numerous studies based on the CHARLS database have been published, with a significant number focusing on sarcopenia. A PubMed search reveals that over 94 sarcopenia‐related articles based on CHARLS data have been published to date, with the majority appearing in the past 3 years. Our journal has also contributed to this body of literature by publishing several of these studies [1, 2]. Although these studies have contributed significantly to the field, we argue that the estimation of ASM in many of these studies may be based on unreliable methods, potentially leading to questionable conclusions.

Sarcopenia is defined by the progressive loss of skeletal muscle mass and function, which significantly impacts physical performance and increases the risk of adverse health outcomes such as falls, fractures and disability. The diagnosis of sarcopenia typically relies on the assessment of ASM, muscle strength and physical performance. The Asian Working Group for Sarcopenia (AWGS) and the European Working Group on Sarcopenia in Older People (EWGSOP) have established consensus criteria for sarcopenia diagnosis, with ASM being a key component [3, 4]. The gold standard for measuring ASM is dual‐energy x‐ray absorptiometry (DXA), which provides precise and reliable quantification of muscle mass [3, 4]. However, in the context of large‐scale epidemiological studies, direct measurement of ASM using DXA or other imaging techniques is often impractical due to cost and accessibility constraints. However, in the context of CHARLS, direct measurements of ASM were not conducted (https://charls.pku.edu.cn/). Instead, many studies have employed an anthropometric formula to estimate ASM, which is derived from a small‐sample study (763 individuals) published in 2011 [5]. This formula is

$$\begin{array}{c} \mathrm{ASM}=0.193\times \text{weight}\;\left(\mathrm{kg}\right)+0.107\times \text{height}\;\left(\mathrm{cm}\right)-4.157\times \\ \;\mathrm{sex}\;\left(1\;\text{for male};2\;\text{for female}\right)-0.037\times \mathrm{age}\;\left(\mathrm{yr}\right)-2.631 \end{array}$$

The accurate estimation of ASM is crucial for the diagnosis of sarcopenia and the assessment of its prevalence and impact. Although this formula has been widely used, we contend that its application in CHARLS‐based studies may be inappropriate for several reasons.

First, the original study from which this formula was derived was conducted in a relatively young population with a mean age of only about 40 years (39.3 ± 14.5 years for males; 41.1 ± 14.1 years for females; 18–69 years old). In contrast, sarcopenia research primarily focuses on older adults, and the physiological characteristics of muscle mass in younger individuals may not be representative of those in the elderly. The sarcopenia research based on CHARLS usually excludes individuals younger than 60, with a mean age of more than 67 [1, 2]. This age discrepancy can significantly impact the applicability and accuracy of the derived formula in sarcopenia research.

Second, the original study was conducted in 2006, whereas many CHARLS data used in recent studies were collected around 2015 [1]. According to data from the General Administration of Sport of China (https://www.sport.gov.cn/n315/index.html), significant changes in the average height and weight of the Chinese population have occurred over the past two decades. These changes may affect the accuracy of ASM estimation using the original formula.

Another critical limitation of the widely used formula is its reliance on only a few anthropometric measurements—height, weight, sex and age—while neglecting other important body composition indicators such as waist circumference and calf circumference. This oversimplification can lead to substantial bias in the estimation of ASM. For example, using a fixed cut‐off point based on the lowest 20th percentile of ASM, as seen in many CHARLS‐based studies [1, 2], may incorrectly classify individuals with low stature and average weight as having low ASM. This approach essentially labels a fixed proportion of the population as having low muscle mass, regardless of their actual muscle status. The exclusion of other relevant anthropometric measurements and the use of a population‐specific cut‐off point without proper validation can result in misclassification and unreliable conclusions regarding sarcopenia prevalence and its associated health outcomes. However, the original CHARLS data collection did not include comprehensive anthropometric measurements such as calf circumference or other indicators that could provide a more nuanced understanding of body composition.

Lastly, the original formula has not been independently validated in other populations, particularly among older adults. Given the potential for population‐specific differences in body composition and muscle mass distribution, the extrapolation of this formula to the CHARLS cohort may introduce significant bias. For example, studies have shown that the relationship between anthropometric measurements and muscle mass can vary by ethnicity, age and sex [4]. Therefore, the use of a formula derived from a different population and time period may not accurately reflect the ASM in the CHARLS cohort.

In conclusion, we urge caution in interpreting studies that estimate ASM using the aforementioned formula in CHARLS‐based research. Future studies should consider validating or updating the ASM estimation methods to better reflect the characteristics of the CHARLS population. We believe that addressing this issue will enhance the reliability and validity of sarcopenia research based on CHARLS data.

## Conflicts of Interest

The authors declare no conflicts of interest.

## References

[1] Y. Hu , W. Peng , R. Ren , Y. Wang , and G. Wang , “Sarcopenia and Mild Cognitive Impairment Among Elderly Adults: The First Longitudinal Evidence From CHARLS,” Journal of Cachexia, Sarcopenia and Muscle 13, no. 6 (2022): 2944–2952. 36058563 10.1002/jcsm.13081 PMC9745544

[2] Y. X. Luo , X. H. Zhou , T. Heng , et al., “Bidirectional Transitions of Sarcopenia States in Older Adults: The Longitudinal Evidence From CHARLS,” Journal of Cachexia, Sarcopenia and Muscle 15, no. 5 (2024): 1915–1929. 39001569 10.1002/jcsm.13541 PMC11446714

[3] L. K. Chen , J. Woo , P. Assantachai , et al., “Asian Working Group for Sarcopenia: 2019 Consensus Update on Sarcopenia Diagnosis and Treatment,” Journal of the American Medical Directors Association 21, no. 3 (2020): 300–7 e2. 32033882 10.1016/j.jamda.2019.12.012

[4] A. J. Cruz‐Jentoft , G. Bahat , J. Bauer , et al., “Sarcopenia: Revised European Consensus on Definition and Diagnosis,” Age and Ageing 48, no. 1 (2019): 16–31. 30312372 10.1093/ageing/afy169 PMC6322506

[5] X. Wen , M. Wang , C. M. Jiang , and Y. M. Zhang , “Anthropometric Equation for Estimation of Appendicular Skeletal Muscle Mass in Chinese Adults,” Asia Pacific Journal of Clinical Nutrition 20, no. 4 (2011): 551–556. 22094840

